# Group A Streptococcus in the Gynecologic Patient

**DOI:** 10.1155/S1064744997000719

**Published:** 1997

**Authors:** P. Garvey, W. J. Ledger

**Affiliations:** 1 Department of Obstetrics and Gynecology The New York Hospital–Cornell Medical Center New York NY USA; 2 NYH-CUMC 525 East 68th St., J-130 New York NY 10021 USA

## Abstract

*Background:* Over the past few decades, physicians have been reminded of the potential for serious complications arising from group A streptococcal (GAS) infections. These infections continue to pose a serious threat, primarily because the pathophysiology of these infections is complex. This article reviews some of the features of GAS infections and presents two case reports of GAS pelvic infections in women.

*Case:* The two patients discussed both had unusual presentations for pelvic inflammatory disease. In both cases, there was strong concern that a serious gastrointestinal process was occurring. Both improved dramatically after aggressive irrigation of their abdominal cavities and administration of antibiotics.

*Conclusion:* Appropriate and aggressive use of antibiotics and reduction of bacterial load through debridement and irrigation are crucial in treating serious GAS infections.


Infectious Diseases in Obstetrics and Gynecology 5:391–394 (1997)
© 1998 Wiley-Liss, Inc.

### Group A Streptococcus in the Gynecologic Patient

**P. Garvey* and W.J. Ledger**

*Department of Obstetrics and Gynecology, The New York Hospital–Cornell Medical Center, New York, NY*

ABSTRACT

***Background:*** **Over the past few decades, physicians have been reminded of the potential for serious complications arising from group A streptococcal (GAS) infections. These infections continue to pose a serious threat, primarily because the pathophysiology of these infections is complex. This article reviews some of the features of GAS infections and presents two case reports of GAS pelvic infections in women.**

***Case:*** **The two patients discussed both had unusual presentations for pelvic inflammatory disease. In both cases, there was strong concern that a serious gastrointestinal process was occurring. Both improved dramatically after aggressive irrigation of their abdominal cavities and administration of antibiotics.**

***Conclusion:*** **Appropriate and aggressive use of antibiotics and reduction of bacterial load through debridement and irrigation are crucial in treating serious GAS infections. Infect. Dis. Obstet. Gynecol. 5:391–394, 1997.** © 1998 Wiley-Liss, Inc.

KEY WORDS
Group A streptococcus; pathogens; pelvic infections

Obstetrician-gynecologists have always been fascinated with group A streptococcus (GAS). Historically, the clinical awareness of the contagiousness of puerperal sepsis was initiated by the studies of Ignatz Semmelweiss in Vienna,[1] and the responsible bacterial pathogen was undoubtedly GAS. In the late 1920s, before the use of antibiotics, an epidemic of GAS infections in a maternity unit in New York City caused serious morbidity and mortality.[2] Although these infections remained serious, the introduction of antibiotics brought this syndrome under control, and these infections became rare among obstetric-gynecologic patients. Recently, there have been reports that the incidence of GAS infections is increasing again.[4, 5]

We want to report two recent cases of peritonitis resulting from GAS infections in gynecologic patients. These women were seriously ill; most of the clinicians caring for these women were not aware of the possibility of GAS infection, for this type of infection had not been seen in our practice for over a decade. These case reports are meant to alert physicians to this diagnostic possibility.

### CASE REPORTS

### Case 1

L.S. was a 41-year-old female gravida 2, para 2, whose last menstrual period began two weeks prior to admission and who developed malaise, fever, and rigors the night prior to admission. The following morning, the patient noted the sudden onset of diffuse abdominal pain. Prior to admission, she had noticed a thick, clear, odorless cervical discharge and dyspareunia for one week and a rectal discharge for one month. Her children had been recently treated for pharyngitis, and both the patient and her husband had been experiencing sore throats. She was in a monogamous relationship, had no history of pelvic inflammatory disease, and had a remote history of herpes.

Upon examination in the emergency room, clinicians found that her oral temperature was 39°C,

*Correspondence to: Dr. Padmavati Garvey, NYH-CUMC, 525 East 68th St., J-130, New York, NY 10021.

Received 17 October 1997
Accepted 13 February 1998

her blood pressure was 80/50 mmHg, and her pulse was 124 bpm. Her abdomen was moderately tender with no localizing signs. She demonstrated guarding but no rebound tenderness. A thick green cervical discharge was seen when L.S. was examined with a speculum, but no rectal discharge was observed. Extreme cervical motion tenderness was elicited by bimanual pelvic examination. Her white blood cell count was 23,000 cells/ml, her creatinine level was 1.2 mg/dl, and a pregnancy test was negative.

A computerized tomographic scan (CT) of her abdomen and pelvis revealed diffusely thickened omentum; hyperemic, mildly enlarged retroperitoneal lymph nodes; edematous broad ligaments; and thickened fallopian tubes, but no abnormal fluid or gas collections in the endometrial canal that would suggest endometritis. Intravenous gentamicin (a loading dose of 120 mg and a maintenance dose of 80 mg every 8 hours) and clindamycin (900 mg every 8 hours) were prescribed for presumed pelvic inflammatory disease.

Over several hours, L.S.'s abdominal examination worsened, and she had had intermittent bouts of hypotension that resolved spontaneously. A repeat pelvic examination again showed cervical motion tenderness but no adnexal masses. A microscopic examination of vaginal secretions showed a field loaded with white blood cells. The patient underwent emergency laparoscopy. Intraoperatively, 100 ml of thick, green fluid was seen in the cul de sac. The serosa over the uterus and tubes was hyperemic. The tubes and fimbrae were thoroughly irrigated and then gently massaged. No purulent discharge was seen extruding from the tubes. No other abnormalities were identified. The pelvis and abdomen were thoroughly irrigated. The patient was given ampicillin (2 g every 6 hours), gentamicin (80 mg every 8 hours), and flagyl (500 mg every 6 hours) for an additional five days. She improved dramatically. A gram stain of pelvic fluid showed polymorphonuclear cells but no bacteria. Blood cultures and cervical cultures obtained from the day of admission showed gram-positive cocci in chains that were eventually identified as GAS. Cervical cultures for gonorrhea and chlamydia were negative. The peritoneal fluid obtained at the time of laparoscopy had no growth.

### Case 2

L.K. was a 39-year-old pediatrician, gravida 4, para 1031, who was admitted to the emergency room four weeks after a normal spontaneous vaginal delivery at term. Her antepartum course was uncomplicated except for a vaginal culture at 36 weeks which was positive for group B streptococcus. The patient received ampicillin prophylactically during labor. Three weeks postpartum, the patient reported vaginal irritation and was treated for a presumed yeast infection. She also recalled having enlarged cervical lymph nodes and a sore throat.

On the day of admission, the patient was evaluated in the emergency room in response to reports of one day of intense abdominal pain, diarrhea, nausea, vomiting, and fever. She was noted to have a temperature of 39°C, a blood pressure of 123/65 mmHg, and a pulse of 113 bpm. She had moderate right lower quadrant tenderness and rebound tenderness in response to abdominal palpation. Her white blood cell count was 11,400 cells/ml. She had cervical motion tenderness and mild uterine fundal tenderness. Vaginal discharge was primarily lochia. Pelvic sonography showed a fluid-filled cecum and ascending colon. A CT scan showed no evidence of endometritis but did show fluid posterior to and inferior to the cecum.

The patient underwent a laparotomy for presumed appendicitis. Intraoperatively, 75 ml of purulent material was seen and thoroughly irrigated. Her appendix, fallopian tubes, and ovaries appeared normal. The patient had negative cervical and peritoneal cultures but had blood cultures that grew GAS. The patient was given ampicillin (2 g every 6 hours), gentamicin (a loading dose of 120 mg, followed by a maintenance dose of 80 mg every 8 hours), and flagyl (500 mg every 6 hours) for seven days. She improved dramatically.

### DISCUSSION

Some recent reports suggest that the incidence of streptococcal infections is increasing.[4, 5] The reason for this trend is not entirely clear. It is possible that the availability of newer antibiotics has decreased the incidence of GAS infections in childhood but has created a population of adults who may be more susceptible to GAS infections due to lack of exposure as children. It is known that varicella infections during childhood are much less severe than those during adulthood. If GAS infections are acquired by adults, they may have a greater potential for serious sequelae if the patient had not had exposure to the organism as a child. Differences in the immune system may be responsible for the differences in severity. This hypothesis will need to be studied to find new ways to treat serious GAS infection. Streptococcal infections and their sequelae can involve a number of sites and can range in severity from a mild, self-limited course to a toxic-shock-like syndrome (TSLS). Common primary sites of infection include the respiratory tract, musculoskeletal system, and skin. In the female reproductive tract, GAS infections are often associated with foreign bodies or recent trauma to the endometrium. Vaginal and anal carriers of GAS in adults are rare.[6, 7] Reports of salpingitis exist in the literature in which no foreign body use, delivery, or instrumentation can be documented.[6, 8] It has been postulated that menstruation may cause enough of a disruption to enable ascending GAS infection in these women. Primary peritonitis secondary to GAS has been described. The diagnosis depends on isolating the organism in the abdominal cavity with no other identifiable source. Salpingitis would be difficult to distinguish from primary peritonitis without obtaining an endometrial and endosalpingeal biopsy. Toxic-shock-like syndrome is the most serious sequelae associated with GAS infections. In such cases, profound hypotension and multisystem organ failure develop rapidly, and there is a 25% mortality rate. In fact, the rapidity with which the two patients described developed their symptoms and the severity of their symptoms are key features of GAS infection.

Despite extensive study, GAS infections continue to pose a serious threat, primarily because of the complex pathophysiology of these infections. The factors that determine the severity of the infection or the sequelae have not been completely elucidated, but evidence indicates that different strains of GAS have different virulence and different interactions with the host immune system. The M protein is the main virulence factor and inhibits phagocytosis. There are 80 different serotypes of the M protein. The GAS cultures isolated from patients with TSLS symptoms possess the M1 and M3 serotypes, as well as newly discovered superantigens. These newly discovered antigens have been labeled streptococcal pyogenic exotoxins A, B, and C. Research indicates that these antigens induce significant T-cell proliferation and initiate the production of large quantities of tumor necrosis factor, interleukins, and various cytokines. The ensuing cascade of powerful immune mediators leads to drastic and widespread tissue damage and shock. Unlike other antigens, superantigens bypass normal antigen processing mechanisms and interact with T cells directly. In addition, most antigens stimulate small populations of T cells because the recruitment of such populations is based on antigen/T-cell compatibility involving five regions of the T cell receptor. Superantigens are able to stimulate up to half the T-cell population because compatibility in only one region on the T-cell receptor is necessary.[9, 10] Although serious GAS infections may be more of a toxin problem than an organism problem, reducing the production of the toxin is key to the management.

The two cases described illustrate how difficult it can be to diagnose group A streptococcal infections in a gynecologic setting. Both patients appeared to have peritonitis. Their clinical courses are significant for the rapidity with which their symptoms developed and the lack of any clear antecedent event. Patient two had recently delivered and endometrial disruption secondary to partition is presumably the manner through which GAS endometritis develops. However, no evidence of an ascending pelvic infection was found. Both patients possibly had prolonged exposure to large reservoirs of GAS since patient one was a mother of several children with pharyngitis and patient two was a pediatrician. Studies have been performed which have confirmed the transmission of Streptococcus pyogenes causing TSLS among family members; based on these findings, antibiotic prophylaxis for close contacts of patients with TSLS has been proposed.[11]

For the practicing clinician dealing with GAS infections, two clinical techniques are important: appropriate and aggressive use of antibiotics and reduction in bacterial load, either through irrigation or debridement. A GAS infection is sensitive to penicillin, clindamycin, and erythromycin. However, the Eagle phenomenon can occur in GAS infections. The Eagle phenomenon is a decrease in penicillin-binding proteins on the wall of GAS with increasing colony counts. Thus, if the bacterial load is heavy, penicillin loses its effectiveness. Therefore, clindamycin may be a better choice, because its effectiveness does not depend on colony size.[9] Early recognition of a possible serious GAS infection is vital since rapid deterioration can occur.

### REFERENCES

1. Semmelweiss IP: The Etiology, the Concept and the Prophylaxis of Childbirth Fever. Translated by F.R. Murray. Baltimore: Williams & Wilkins, 1941. Originally published in 1861.
2. Watson BP: An outbreak of puerperal sepsis in New York City. Am J Obstet Gynecol 16:157–179, 1928.
3. Ledger W, Headington J: Group A β-hemolytic streptococcus. Obstet Gynecol 39:474–482, 1972.
4. Weiss K, Laverdiere M: Group A streptococcus invasive infections: a review. Can J Surg 40:18–25, 1997.
5. Demers AE, Simor AE, Vellen PM, et al.: Severe invasive group A streptococcal infections in Ontario, Canada: 1987–1991. Clin Infect Dis 16:792–800, 1993.
6. Fikrig E, Worthington M, Lefkowitz L: Septic shock and acute respiratory distress syndrome after salpingitis caused by streptococcus pyogenes group A. Southern Med J 82:634–635, 1989.
7. Barham W, Haberberger R, Decker C: Group A streptococcal sepsis secondary to acute salpingitis. Clin Infect Dis 16:444, 1993.
8. Brown-Harrison M, Christenson J, Harrison M, Matlak M: Group A streptococcal salpingitis in a prepubertal girl. Clin Pediatr 556–558, 1995.
9. Stevens D: The toxic shock syndromes. Infect Dis Clin North Am 10:727–747, 1996.
10. Noronha S, Yue C, Sekosan M: Puerperal group A betahemolytic streptococcal toxic shock-like syndrome. Obstet Gynecol 88:728, 1996.
11. Ichiyama S, Kazumitsu N, Shimokata K, et al.: Transmission of Streptococcus pyogenes causing toxic shocklike syndrome among family members and confirmation by DNA macrorestriction analysis. J Infect Dis 175:723–726, 1997.
